# Efficacy of S-PRG filler containing varnishes on enamel demineralization prevention

**DOI:** 10.1038/s41598-020-76127-w

**Published:** 2020-11-04

**Authors:** Manuela da Silva Spinola, Sabrina Elise Moecke, Natália Rivoli Rossi, Toshiyuki Nakatsuka, Alessandra Bühler Borges, Carlos Rocha Gomes Torres

**Affiliations:** 1 Department of Restorative Dentistry, São Paulo State University - UNESP, Institute of Science and Technology, Av. Eng. Francisco Jose Longo, 777, São José dos Campos, SP 12245-000 Brazil; 2 Department of Dental Materials and Prosthodontics, São Paulo State University - UNESP, Institute of Science and Technology, Av. Eng. Francisco Jose Longo, 777, São José dos Campos, SP 12245-000 Brazil; 3 Department of Research and Development, Shofu Inc., 11 Kamitakamatsu-cho, Fukuine, Higashiyama-ku, Kyoto, 605-0983 Japan

**Keywords:** Health care, Medical research

## Abstract

This study evaluated the efficacy of S-PRG vanishes on preventing enamel demineralization. Bovine enamel specimens were obtained, polished and the baseline Knoop microhardness was evaluated. Specimens were stratified into six groups (n = 15), according to the varnish applied: S10—experimental varnish containing 10% of S-PRG fillers, S20—20% of S-PRG fillers, S30—30% of S-PRG fillers; S40—40% of S-PRG fillers; PC (positive control)—5% of NaF; NC (negative control)—no treatment was performed. Half of enamel surfaces were protected to work as a control and varnishes were applied over the unprotected area. A demineralizing pH-cycling was performed, and surface and cross-sectional microhardness were measured. The percentage of microhardness of the treated area was calculated comparing with the untreated area. Statistical analysis was performed by one-way ANOVA and Tukey’s test (p = 5%). All experimental S-PRG varnishes protected against demineralization in relation to no treatment, but S40 was the most effective on the surface. For all depths, S30 and S40 were superior in enamel demineralization prevention than other S-PRG filler concentrations and 5% NaF. It was concluded that S-RPG filler containing varnishes were effective to prevent enamel demineralization. The higher concentrated products were more effective than 5% sodium fluoride on surface demineralization prevention.

## Introduction

Varnishes are by definition coat materials that forms a thin layer on the surfaces after application, creating a solid film after physical or chemical changes, working as a functional coating. In dentistry, the functional properties of the varnishes are related to the remineralization, antimicrobial and bleaching effects. They are generally a dispersion or solution of resin, monomers or polymers (binding agent) and addictive (active and auxiliary ingredients) in a liquid medium (solvent)^1,2^. Despite the type, all varnishes contain film formers. Its interaction with the surface, environmental conditions and active ingredients determine the characteristics of the varnish. They provide a simple, reliable and quick topical application, and delayed and slow release of active compounds^1^.

As the varnish is a carrier of active ingredients, its layer should bond to the surface until they have been released on the surface, acting as a “release device”^1^. The formation of a solid layer and slow releasing of fluoride varnishes are the main difference in relation to other regular fluoride applications methods, such as gels or foams. According to the curing mechanism, the varnishes can be classified in physically, chemically of light-cured. For the first one, the curing occurs by the evaporation of the solvent, for the second by a chemical reaction and for the last after exposure to a light source.

It was shown by previous researches that fluoride agents in high concentration, such as sodium fluoride gels or varnishes, are effective for helping to prevent the formation and to promote the remineralization of enamel caries lesions^3–6^. Physically cured fluoride varnishes were developed as an effort to improve the former vehicles for topical fluoridation, such as gels or mouthrinses, prolonging the contact of fluoride ions with the enamel surface^3^. Previous studies showed that the use of 5% fluoride varnish (Duraphat, Colgate) promote significant caries lesions prevention^5,7–9^, as well as white spot lesion remineralization^4–6^. However, some studies showed that, after fluoride varnish application, although the mineralization increase on the more external areas of the lesion, it does not occur in deeper regions of the lesion body. The decrease of porosity on the lesion surface may prevent ions precipitation in deeper areas. It is expected that ion precipitation in deeper layers would provide a more stable and effective remineralization^10,11^. That is even more important for those severe and deeper lesions, and better alternatives still need to be investigated^12^. Furthermore, two systematic reviews showed that more clinical studies are needed to prove that fluoride varnishes application directly influences on decrease of caries incidence^13,14^ and prevention of demineralization process.

As an alternative to protect the tooth structure from caries lesion formation and progression, products containing bioactive components are promising and have been incorporated into different dental materials^15–17^. One of these bioactive components is the surface pre-reacted glass-ionomer filler (S-PRG filler)^18^. For its production, a special fluoro-boro-aluminosilicate glass is prepared by melting the mixture of silica, mullite, boric oxide, cryolite, strontium fluoride and strontium carbonate, which is subsequently milled^19^.

The surface is treated with a polysiloxane solution, creating a porous external silica-glassy layer. To produce the reaction of the glass filler, they are sprayed with a polyacrylic acid aqueous solution, creating a stable pre-reacted phase between the external silica-glassy layer and the inner glass core, which is capable to release six different ions^18^. The S-PRG filler is incorporated in different resin-based restorative materials, creating bioactive properties. Its incorporation in composites have been reported as an efficient inhibitor of dental plaque formation^20^, and enamel^21^ and dentin demineralization^22^, preventing secondary caries^23–25^.

The S-PRG filler releases the ions fluoride, aluminium, borate, strontium, sodium and silicate^19^. Each ion has its respective function, but they can show a synergic effect on caries lesions prevention. Fluoride acts to reduce demineralization by changing the critical pH and promoting the deposition of fluorapatite, fluoridated apatite and calcium fluoride-like precipitates. The aluminium forms alumino-fluoro complexes^23,24^, which help to decrease the process of enamel demineralization and promote remineralization of initial carious lesions^17^. The borate presents antibacterial effect^25^, reducing biofilm formation, and might decrease demineralization. Strontium converts hydroxyapatite in strontiumapatite, which increases acid resistance and enhance the mineralization process^15,17,26^. It also leads to bacteria growth inhibition^25^ and caries prevention^27^. The silicate induces remineralization, by inducing apatite nucleation on the tooth surface^28,29^. The ions Sr^2+^, Na^+^, Al^3+^ act as a strong base and have an acid buffer effect, neutralizing the bacterial acid and protecting against demineralization^26^. Based on that properties, the manufacturer developed a light-cured S-PRG varnish, in order to allow the effect of those ions on the desired tooth surface. Some studies showed its effectiveness on enamel remineralization and protection, as well on root caries prevention^26,30,31^.

However, the light-curing varnish requires the use of a light source, and the resin component can create a certain color change while in place, as well suffer some staining in some situations. That has stimulated the manufacturer to create experimental physically cured clear varnish, similar to the idea of fluoride varnishes frequently used. However, the new formulation creates a mechanically stronger film, with high bonding to the tooth surface by the incorporation of a bioadhesive molecule, intended to stay for longer periods attached to the caries affected/susceptible area. In this case, the multi ion release may be more effective on caries lesion prevention than just the fluoride ions available from the regular varnishes, improving the protective effect. Although there are some previous evaluations of the light-curing S-PRG varnish, there are no previous studies about the potential of the new physically cured varnish formulations on the enamel protection against demineralization.

Thus, the objective of this study was to evaluate the effect of varnishes containing different concentrations of S-PRG filler on the protection against enamel demineralization. The null hypothesis tested was that the experimental S-PRG varnishes do not significantly protect the tooth enamel against demineralization.

## Methods

### Specimen preparation

Intact bovine incisors were obtained from recently slaughtered animals with an average age of 3 years. A total of 118 teeth without any visual signs of cracks, caries or non-carious lesions were used. Cylindrical specimens (6 mm of diameter) were obtained from labial surface of bovine incisors, using a diamond trephine mill in a circular cutting machine under water cooling. Previous studies showed that the mineral content of enamel is reduced from the tooth surface toward the DEJ^32,33^, which can have an effect if the enamel close to the surface is compared with that on deeper areas^34^. Therefore, only 100 µm of the external enamel was removed to produce a flat surface, allowing the cross-sectional microhardness to be performed in the underlying 100 µm, within an area where the previous studies suggested that change of mineral content is small^32,33^. The removal of enamel surface also eliminated the tissue that contains relatively high concentration of artificially introduced trace elements due to the contact with the saliva^35^. For that, the specimens were placed inside the slot of a metal holder, which was basically a stainless-steel cylinder with a central hole filled by an internal screw to adjust the depth of the slot. The original enamel surface was left 0.1 mm outside the holder surface adjusting the screw. The outer enamel was removed using a P1200 SiC sandpaper (Extec Corp., Enfield, CT, USA) coupled in an automatic polishing machine (Panambra, Sao Bernardo do Campo, SP, Brazil). After that, the depth of the slot was adjusted to 2 mm and the specimen was placed inside with the enamel toward the screw. The excess of dentin was removed with P1200 sandpaper, standardizing the thickness of the specimens in 2 mm. The enamel surface was again placed outward and polished using a sequence of SiC sandpapers grits P2400 and P4000 (Extec Corp., Enfield, CT, USA), for 60 and 120 s, respectively. Ultrasound bath was performed after the use of each sandpaper for 10 min to remove abrasive residues. The total area of the polished enamel surface was 28.274 Mm^2^. The prepared specimens were examined under the stereomicroscope (Discovery V20, Karl Zeiss, Jena, Germany), using a magnification of 25 ×, to certify the absence of cracks, hypomineralized (white spot) areas or other surface defects. A minimum baseline microhardness threshold was set for the intact enamel. For that, specimens with a Knoop Hardness Number (KHN) smaller than 300 were excluded, resulting in 90 specimens in perfect conditions. The specimens were placed inside individual silicone holders, which were kept inside a plastic container with 100% of relative humidity, in a way that the enamel surface had no contact with water, which could cause surface alterations of the original hardness^35^. The study was started within one week after finishing the specimens.

### Baseline Knoop microhardness

**Figure 1:**
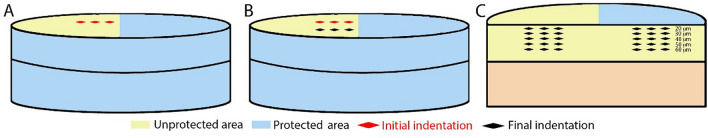
(**A**) Baseline surface Knoop microhardness analysis on the unprotected enamel; (**B**) surface Knoop microhardness after pH cycling; (**C**) cross-sectional Knoop microhardness analysis performed on the unprotected and protected enamel after pH cycling.

Baseline Knoop microhardness was evaluated using a microhardness tester (FM -700, Future-Tech Corp, Tokyo, Japan) with an automatic X–Y stage system (ARS 900, Future-Tech Corp), which allowed the precision and sequential measurements, by inputting the parameters in the microhardness analysis software (FM-ARS 9000, Future-Tech). Each indentation was obtained by using a load of 25 g and 5 s dwell time and the results were expressed as KHN^12,36,37^. Three indentations were performed on enamel surface, with a distance of 100 µm among them by using the software, and the mean value was calculated. The mean of the baseline KHN was 362.67 (± 26.34). After that, the lateral and the lower surfaces were protected with an acid-resistant nail polish (Cora, LBE, Serrana, SP, Brazil), containing butyl acetate, ethyl acetate, nitrocellulose, acetyl tributyl citrate, isopropyl alcohol, pigment, hydrated silica and acrylate copolymer. This nail varnish has no effect on caries lesion formation and was used only as mechanical and impermeable barrier to protect the areas not treated from the effects of the pH cycling. It was completely insoluble to water, remaining during the whole time of the study. In addition, half of the enamel surface was also protected with the nail polish and used as a control of the intact enamel microhardness on the posterior stage of cross-sectional microhardness test. The area of the polished enamel actually exposed to the treatment was 14.137mm^2^ as can be seen in Fig. 1. After application of the nail polish, the specimens were placed back inside the plastic container with 100% of relative humidity for 24 h, allowing the complete evaporation of the solvents and full setting of the layer, before starting the treatments, creating an impermeable protection.

### Experimental groups division and treatment

The specimens were stratified into six different groups (n = 15), according to the baseline surface microhardness values, in order to have similar initial KHN means. The groups received the following treatments:

- NC (negative control)—the specimens did not receive any treatment;
- PC (positive control)—the specimens received the application of an experimental varnish containing 5% of sodium fluoride (w/w);
- S10—the specimens received the application of an experimental varnish containing 10% of S-PRG fillers (w/w);
- S20—the specimens received the application of an experimental varnish containing 20% of S-PRG fillers (w/w);
- S30—the specimens received the application of an experimental varnish containing 30% of S-PRG fillers (w/w);
- S40—the specimens received the application of an experimental varnish containing 40% of S-PRG fillers (w/w);

All varnishes have the same formulation in relation to the biding agent, auxiliary ingredient and solvent, to which were added the active component. The basic composition of the varnishes was ethanol (solvent), colophonium resin (biding agent), bioadhesive (auxiliary ingredient) and S-PRG filler or sodium fluoride (active ingredients). The mean particle size of S-PRG filler was 0.8 μm. All varnishes tested were produced by Shofu Inc (Kyoto, Japan), and were not commercially available and have not a commercial name.

The varnishes were provided inside a small plastic blister, which has a cavity containing the product, and a peel-open lidding foil to seal the cavity and avoid interaction of the product with the atmosphere. The S-PRG particles or sodium fluoride salts were already mixed with the other ingredients inside the blister. However, due to the force of gravity, the particles were settled in the bottom of the cavity. The manufacturer also provided a standard plastic brush for the varnish application. Before using, the lidding foil was removed and the varnish properly mixed using rotary movements for 5 s, creating a suspension of the active ingredients. A new and clean brush was dipped into the varnish for 1 s, removed from the cavity and immediately applied to the specimens, over the unprotected area of the surface enamel. As the specimens had a round surface and were half covered with the protector nail polish, the brush touched one edge of the unprotected area and was gently moved toward the opposite side. Just a single application was performed on each specimen by the same operator over all samples. Before the application, the operator trained many times until reaching a standard procedure. In order to determine the amount of varnish applied, the specimen’s weight was measured before and after the application using an analytical balance (Excellence XP204, Mettler-Toledo AG, Greifensee, Switzerland). The mean weight of the varnish applied per specimen was 0.87 mg. All the varnishes had the same appearance and packaging, and were codified by a person not involved in the study, using letters A, B, C, D, and E. Therefore, the operator was blinded to the products applied on each group and during microhardness analysis. The codes were broken only after statistical analysis. Immediately after the application^13,38^ the specimens were immersed into artificial saliva at 37 °C for 24 h, using the formulation proposed by Klimek et al.^39^. The varnishes remained on the surface during the next phase of pH cycling.

### Demineralizing pH-cycling

In order to produce artificial caries lesions, a pH cycling specifically developed to produce early carious lesions in bovine enamel, avoiding dental erosion, was performed using demineralizing and remineralizing solutions^37^. The demineralizing solution contains 1.28 mM Ca, 0.74 mM P, 0.03 µg F/ml and pH 5, resulting in 50% undersaturation when compared to enamel^37^. Remineralizing solution contained 1.5 mM Ca, 0.9 mM P, 150 mM KCl, 0.05 µg F/ml, 0.1 M Tris buffer and pH 7, resulting in 100% saturation in relation to enamel^37^. The specimens were immersed in demineralizing solution for 4 h and then 20 h in remineralizing solution^37^. A ratio of 6.25 ml/mm^2^ and 3.12 ml/mm^2^ was used for the demineralizing and remineralizing solution in relation to the unprotected surface area exposed to the treatments, respectively^37^. The pH cycling was performed for 8 days^37^. After that, all specimens were cleaned with cotton wool soaked in acetone, in order to remove any varnish residues from the surface and allow the final surface microhardness evaluation.

### Final microhardness measurement

Surface and cross-sectional microhardness were measured after treatments and pH-cycling regimen, so it was possible to evaluate varnish protection against demineralization comparing with the baseline and control measurements. Surface microhardness measurement of the treated area was performed close to the baseline reading (Fig. 1B). The fact that the baseline indentations were visible after the pH cycling indicated that no surface loss occurred. Then, the specimens were embedded in acrylic resin (Jet, Classico, São Paulo, SP, Brazil), ground perpendicularly to the limit line between the protected (untreated) and unprotected surface (treated). The transverse cut area was polished with SiC sandpapers similarly to what was performed on the surface.

The cross-sectional microhardness was measured from the enamel surface toward the dentin-enamel junction. Five depths were analyzed, at distances of 20, 30, 40, 50 and 60 µm from the surface. The constant interval of indentation was performed by the microhardness tester using the automatic X–Y stage system, that was pre-programmed to make 5 indentations in the same direction, perpendicularly to the enamel surface, with 10 µm distance between them. The first distance measurement of the 20 µm from the surface was calculated based on the automation software of the device. For each depth, three indentations were performed on the treated and additional three on untreated area (Fig. 1C). A mean value for each depth was calculated. The percentage of Knoop microhardness (%KHN) of the treated area in relation to the untreated region (considered 100%), for the same depth, was calculated using the following formula: %KHN = (KHN_treated_ × 100)/KHN_untreated_.

### Statistical analysis

The normality assumption of the data was evaluated by Kolmogorov–Smirnov test, while the homogeneity of variances was analyzed by Levene's test, before and after the pH cycling. For each depth, the difference among the groups was analyzed using one-way ANOVA and Tukey’s test. For each group, the differences among the various depths were analyzed with the same tests. A significance level of 5% was set for all analysis.

## Results

The Kolmogorov–Smirnov test showed a normal distribution at the baseline (d = 0.0647) and after the treatments (d = 0.0970). Levene's test showed homogeneity of variances before (p = 0.2230) and after the treatments (p = 0.9999). The results of ANOVA for all groups and depths are shown in Table 1. For the comparison among the groups for each depth, significant differences were observed only for surface, 20 and 30 µm. In this case, the control group without treatment showed significantly smaller percentage of final microhardness in relation to the intact enamel than other groups. For the surface, S40 showed significantly higher %KHN than the group in which the fluoride varnish was applied. Non-significant differences were observed among the experimental groups for all the other depths.

**Table 1:** Means (SD) of %KHN for the different depths and results of Tukey’s test.

Groups	DEPTH						ANOVA^•^
	Surface	20 µm	30 µm	40 µm	50 µm	60 µm	
Control	48.24 (8.35) Aa	54.40 (12.03) Aa	77.49 (11.68) Ab	89.44 (8.36) Ac	95.74 (14.63) Ac	99.97 (9.68) Ac	0.0001*
10% S-PRG	84.34 (13.83) BCa	94.71 (15.13) Bab	94.85 (12.07) Bab	97.72 (6.89) Ab	97.05 (7.35) Ab	99.20 (10.58) Ab	0.0093*
20% S-PRG	85.19 (13.19) BCa	95.57 (13.57) Bab	97.15 (6.59) Bb	98.11 (10.94) Ab	100.46 (7.32) Ab	100.04 (11.99) Ab	0.0027*
30% S-PRG	88.68 (16.58) BCa	94.22 (28.08) Ba	94.47 (16.62) Ba	96.38 (18.53) Aa	99.22 (8.14) Aa	100.10 (9.34) Aa	0.5793
40% S-PRG	97.29 (24.46) Ca	94.60 (20.10) Ba	95.11 (9.03) Ba	97.99 (8.82) Aa	99.75 (13.34) Aa	101.12 (14.00) Aa	0.8612
FLUORIDE	79.57 (18.11) Ba	92.81 (22.38) Bab	98.82 (23.68) Bb	100.73 (10.37) Ab	100.34 (6.48) Ab	101.14 (5.45) Ab	0.0023*
ANOVA**	0.0001*	0.0001*	0.0014*	0.1362	0.7392	0.9956	

**Results of ANOVA for the comparison among the groups for each depth; ^•^Results of ANOVA among different depths for each group; *Significant differences. Different capital letters in the columns indicate differences among the groups for each depth. Different lower-case letters indicate differences among the depths for each group.

For the comparison among different depths for each group, significant differences were observed for the control, S10 and S20 and fluoride varnish groups (Table 1). For the control group, the %KHN for surface, 20 and 30 µm were smaller than the other depths. For S10, S20 and PC (sodium fluoride group), the surface values were significantly smaller than 30, 40, 50 and 60 µm. For S30 and S40, non-significant reduction of microhardness was observed of all depths.

## Discussion

Some studies showed that enamel caries lesions are formed faster in bovine than in human enamel^35,40,41^. The difference in dissolution rate between the tissues was shown to be approximately 1.4:1, but both having a linear dissolution behavior^35^. Although the mineral content of sound bovine enamel is not significantly different from human^42^, the hardness of bovine enamel tends to be smaller^43,44^. The differences on dissolution may be explained by differences in microstructure and chemical composition^35^. The bovine enamel has larger crystals^45^ but smaller prims diameters^46^, more interprismatic substance^47^, and higher porosity^45^. A study showed that the carbonate content of bovine enamel (4.2 ± 0.8 wt%) was significantly higher than the human enamel (2.0 ± 0.5 wt%)^48^, while another showed a smaller fluoride content^49^. That creates different pattern of prisms and crystals dissolution, raising concerns about the use of bovine tooth instead of human in caries analysis^50^. The rate of lesion formation is dependent of diffusion of chemical species through the enamel^40^. A study observed that diffusion rates in bovine enamel are 3 times higher than in human enamel. They pointed that the quantitative discussion of artificial lesion formation must be done considering these differences^40^. The higher porosity of the bovine enamel allows a quicker penetration of the acid. Nonetheless, the inherent solubility of the tissues also varied greatly between batches of both bovine teeth, which impact the extent of demineralization^51^. That leaves open the question about the importance of the relative differences between bovine and human tooth enamel^51^ pointed by some particular studies.

However, a study showed a similar response to fluoride between bovine and human enamel^52^, and another found no differences in relation to their response to de- and remineralization procedure^53^. Lippert and Hara concluded that human and bovine enamel exhibited a good fluoride dose–response for remineralization and correlated well^41^. Lippert and Lynch pointed that artificial lesions produced on bovine or human enamel have an almost indistinguishable mineral distribution characteristic^35^. Queiroz et al. showed that the pH-cycling model used in the current study, applied to bovine enamel, was adequate to analyze in vitro the effect of low fluoride concentration on caries progression^37^. Therefore, the differences are quantitative rather than qualitative nature^51^. In the case of the analysis regarding the S-PRG varnishes tested in the current study, it is expected that the demineralization depth of the artificial lesions could be different if the research was performed in human enamel. However, the results about the comparison among the products would be similar.

Specimens were treated with the varnishes and remained in artificial saliva for 24 h before the pH-cycling. This period was chosen because it is known that 5% sodium fluoride varnish, which is considered as the gold standard (and positive control in the current study), is able to reach its maximal reactivity with enamel during this time^54^. After that, specimens were submitted to a demineralizing pH-cycling model, aiming to simulate a clinical cariogenic challenge, in which the demineralization process is higher than the remineralization one, leading to creation of an artificial caries lesion. Therefore, the test evaluated if the varnishes used were able to prevent the enamel demineralization even under this cariogenic protocol^37^.

The pH-cycling model used in this study, including the number of days, was established by Argenta et al.^64^ to human enamel and later adapted for bovine enamel by Queiroz et al.^43^, in order to create set of conditions to produce early carious lesions, that could be evaluated by cross-sectional microhardness measurements, while preserving the enamel surface from erosion, previously observed using other demineralization protocols^63^. Analyzing the data from the control group, which was submitted to the pH cycling, without any varnish application, it is possible to see that after 50 µm depth, the microhardness values were very close to the microhardness of sound enamel. Therefore, the maximum lesion depth should be around that depth. A previous study analyzing the lesion depth using polarized light microscope, after the same the pH-cycling demineralizing model used in the current study, observed the maximum lesion depth of 64 µm for the control groups without treatment, very close the hardness alteration depth showed in the current study^37^. Another research evaluated the lesion depth using microradiography, with the same demineralizing protocol used in this study, and observed a lesion depth of 43 µm^55^. We assumed that the measurement of deeper layers would not bring significant information, considering that the enamel was intact in the maximum depth analyzed.

The regular fluoride varnishes films are not expected to remain longer on the surface but to be quickly removed by brushing and wear. Therefore, the effect on the coated tissues will depend on the retention period and concentration of the active component, as well its release rate^56^. That will be related to the hydrophilic properties and solubility of the individual component, and the interaction between the active component and the binding agent, as well the thickness of the layer formed^1,2^. The binding agent has to interact with the surface and exhibit adhesion to the tooth, by polar or bonding groups, or mechanical retention to the porous caries-affected enamel. As enamel and dentin surfaces are polar, the polar polymers are good film formers. The low-molecular resins bonds to the tooth by van der Waals interactions^1^.

Colophony, the biding agent in the experimental varnishes tested, consists of various resin acids, especially abietic acid. The specific kind of colophony used in the formulation of the experimental varnishes contains a large number of carboxyl group, which react with the strontium ions released by S-PRG filler. According the manufacturer, the strong covalent bond significantly increases the stiffness of the varnish layer, becoming more resistant to the interaction with the oral environmental, such as dissolution, temperature variations and brushing, improving its retention time and capacity to allow the interaction with the caries affected area. In addition, the varnish contains a special polar bioadhesive component (not disclosed by the manufacturer), which strongly bond to the tooth surface, improving the retention of the varnish layer. Therefore, the aim of the manufacturer was to create a durable layer covering the caries susceptible or affected area, higher than the former commercially available materials, to extend the ionic interaction with the tooth tissues. An in vitro study (unpublished) compared the retention of the experimental varnish formulation tested in the current study, after being applied over flat enamel surfaces, in relation to other commercially available products. After 2000 thermal cycles (4 °C–60 °C), they observed that the layer was completely preserved for the experimental material, while for the other varnishes the films were partially or completely lost.

The traditional varnishes contain particles of sodium fluoride salts in the formulation, mixed with the other ingredients. Only a small percentage of the salts are already dissolved into the product, while the rest remains as particles (≅ 80%)^54^. When applied to the tooth structure, the water from saliva penetrate into the varnish layer dissolving the salts, which starts to form calcium fluoride (CaF_2_)-like reservoirs on the enamel, at the interface between the varnish and the tooth. This leads to the degradation and softening of the varnish layer, which will eventually be removed by the salivary flow, contact with soft tissues, food bolus or brushing. To increase the effect of the treatment, the manufacturers recommend delaying eating and prevent brushing on the area for some hours. Despite that, it will certainly be lost is a short time interval, leaving the CaF_2_-like reservoirs, which will slowly release fluoride when the pH is reduced.

The retention time plays a role on the effect of the treatment, but only up to a certain period. A previous study kept 5% NaF varnishes over the bovine enamel surface for 8, 12, 18, 24, 36 or 48 h. There was a linear correlation between remineralization and retention time, but the maximum effect was reached after 18 h, without any improvement afterward^2^. Fernández et al. analyzed the effect of the retention time on the formation of CaF_2_-like reservoirs on the bovine enamel^54^. They found that the maximum concentration of CaF_2_ was reached at 24 h and was stabilized after that. Therefore, longer retention times for 5% sodium fluoride varnishes appears not necessary.

For the S-PRG filler containing varnishes, the scenario is completely different. This particle is basically an insoluble glass, with a thin external modified layer capable to release ions to the environment. Therefore, in contact to the water in the oral environment, the varnish will absorb water and the ions will be released, but without degradation of the filler. This characteristic allows these fillers to be used in the formulation of composite resins, which must remain stable during many years in contact with the saliva. Therefore, the S-PRG filler helps to reinforce the varnish, like in the restorative composite. That makes the film created by the experimental varnish stronger and more stable, in association with the higher bonding to the tooth structure provided by the bioadhesive ingredient. Thus, the idea of the manufacturer was to create a pellicle that could remain for many weeks. Although the brushing can help to remove the varnish, the areas where the tooth brushing have constant contact does not show biofilm growing and are less susceptible to the lesion formation. However, in difficult to reach areas, prone to biofilm accumulation, the varnish may have a longer effect.

In relation to the demineralization preventive effect of the experimental varnishes tested, it is due to synergic effect of the mechanical film covering of the surface with the ionic activity of the S-PRG, as well sodium fluoride in the control. If only the presence of a film covering the surface produced the effect, no differences would be observed among the groups. In our results, it was possible to see differences among the S-PRG concentrations tested and in relation to sodium fluoride (Table 1).

All the experimental varnishes tested presented a protective effect against enamel demineralization when compared to the negative control group, rejecting the null hypothesis. The addition of 40% of S-PRG filler in the varnish showed higher protective effect when compared to 5% sodium fluoride varnish in the enamel surface (Table 1). Besides, the addition of 30% and 40% of S-PRG filler prevented a significant reduction of microhardness on deep areas when exposed to the pH cycling.

Fluoride varnishes containing 5% NaF present a great role in preventing enamel demineralization due to its high F^-^ concentration. The fluoride mechanism consists in a local effect, reacting with enamel surface forming CaF_2_-like calcium fluoride deposits^57^. During the pH drop that occurs many times a day, due to biofilm accumulation over teeth surface, the F^−^ from these deposits are released to the oral environment to interfere with demineralization and remineralization processes. Also, the F^−^ forms fluorapatite, integrated in the crystal lattice of the enamel^58^, even when the fluoride presence in these deposits is in small concentrations.

In the current study, the positive control group (5% NaF) presented demineralization on the subsurface (20 µm), showing that the protection was not enough to completely prevent the mineral loss, which is in agreement with other previous fluoride varnish studies^10,38^. The fluoride uptake after varnish application varies among the different products available on the market, depending on the specific composition and characteristics^56^. The NaF varnish used in this study had the same components of the S-PRG varnishes tested, which allow a proper comparison of the ionic interaction with the tooth structure.

Kataoka et al. evaluated the fluoride release of the experimental varnish containing 40% of S-PRG filler and a similar basic formulation containing 1% of NaF^59^. A circular area with diameter of 6 mm (28.27433 mm^2^) was delimitated on a plastic sheet using an adhesive tape. The varnishes were applied on the delimitated area and the specimen was immersed in 5 ml of deionized water for 3 days at 37 °C. After that, the released fluoride ion was measured using an electrode. The concentration of fluoride detected was 0.8732 ppm for the 40% S-PRG and 1.0126 ppm for the NaF varnish. The ratio between fluoride release versus area was 0.0308831 ppm/mm^2^ for the 40% S-PRG and 0.035816 ppm/mm^2^ for the 1% NaF varnish.

An in vitro study performed by the manufacturer (unpublished results) evaluated the fluoride release of the experimental varnish containing 40% S-PRG filler, besides Clinpro White Varnish (3M/ESPE) and MI Varnish (GC), both containing 5% of NaF. The authors embedded in acrylic resin the crown of bovine incisors and created a flat surface. A circular area with a diameter of 5 mm (19.63495 mm^2^) was delimitated. The specimens were immersed into 5 ml of distilled water for 24 h and the fluoride concentration was measured with an electrode. The fluoride ions concentration obtained was 0.8571 ppm for 40% S-PRG, 2.5 ppm for Clinpro White Varnish and 1.5476 ppm for MI Varnish. The ratio between fluoride release versus area was 0.0436 ppm/mm^2^ for the 40% S-PRG, 0.1273 ppm/mm^2^ for the Clinpro White Varnish, and 0.0788 ppm/mm^2^ for the MI Varnish. If the data of fluoride release provided by the manufacturer was taken into account, for the area delimitated in the current study (14.137 mm^2^), around 0.6163 ppm was released by specimen for the 40% S-PRG varnish.

The findings of this study showed that S-PRG varnishes were efficient to prevent enamel demineralization under pH cycling and that high concentrated S-PRG varnishes were better than the fluoride varnish used on enamel surface. This emphasizes the importance of the different ions released (fluoride, aluminium, boron, sodium, strontium and silicate) on demineralization prevention. Clinical effects of S-PRG filler appear to be promising based on the findings of this and other previous studies^25,26,60–64^. The manufacturer analyzed the ionic release after the application of 40% S-PRG varnish on the enamel, over a circular area with a diameter of 5 mm (19.63 mm^2^). After 10 days immersed in 5 ml of distilled water, the elemental analysis was performed by ICP-AES for each specimen. The means of total elemental release was 11 ppm of Na, 3.86 ppm of F, 0.9 ppm of B, 0.2 ppm of Al, 1.8 ppm of Si and 6.4 ppm of Sr.

The multi-ions released from S-PRG filler have three main actions, which are acid neutralization, mineral deposition and antimicrobial. The acid neutralization occurs by the buffering effect. This buffer effect is promoted by strontium, sodium and aluminum, when released from the S-PRG filler. This characteristic effectively affect the demineralization process and the pH challenge that naturally occur in oral environment^17,23,26^. The manufacturer also compared the acid neutralizing capacity of the 40% S-PRG formulation in relation to Clinpro White Varnish and MI Varnish. The varnishes were applied over a round plastic plate with a diameter of 1.5 mm. The plate was immersed in 5 ml of lactic acid solution (pH 4) at 37 °C for 24 h. After this period, the final pH of the solution was 7.07 for the 40% S-PRG, 1.8 for the Clinpro White Varnish, and 5.10 for the MI Varnish.

In relation to mineral deposition, the ions released by S-PRG filler increase mineral uptake when remineralization occurs; and process is mainly assisted by fluoride, strontium and silicate^15,17,19,26^. The strontium plays an important role on the interaction to hydroxyapatite, forming strontium-apatite^65–68^. When compared to fluorapatite, strontium-apatite provides an enhanced acid resistance^65,69^. The specific reason for this is still unclear, but some studies reported that the formation of a calcium-strontium apatite complex at the apatite crystal surface retards the acid dissolution^65,70,71^. It seems that the strontium concentration interferes directly on the enamel remineralization process, since when in higher concentrations, rapid remineralization was observed^66^. The silicate ions from S-PRG filler promote mineralization by silica gel absorption of calcium and phosphate, from the surrounding environment, which generates heterogeneous apatite nucleation^68^. When nucleated, it forms a bone-like apatite layer^72^, which contributes to dentin mineralization^28^.

In relation to the antimicrobial effects of S-PRG filler, it is due mainly to boron, strontium and fluoride^26,60,61^. Boron is used as antimicrobial agent in different human health products^11^. It acts including against *S. mutans,* and reduces the bacterial growth^15,61,73^ and adhesion to tooth surface^25,61,62,64^. Strontium has considerable antibacterial contribution, especially when associated to fluoride^20,74^. Fluoride disturbs bacterial metabolism and growth^20^. Although not analyzed in the present study, the antibacterial and antifungal effects generated by the S-PRG filler were also observed in different studies^62–64^.

In this study, S40 showed the most favorable results compared to all experimental groups on surface microhardness analysis. This can be associated to the higher availability of fillers and ion release, increasing the interaction with the hydroxyapatite and providing the improved resistance that was mentioned before^16,65,67,69–71^. Regarding the depths, non-significant reduction of microhardness was observed for S30 and S40 in all depths, which means that the demineralization process did not significantly occur. Similar results were found in studies evaluating the S-PRG containing light-curing varnish (PRG-Barrier Coat, Shofu), which has a filler loading of around 30% w/w that efficiently prevented enamel demineralization when compared to NaF varnish^17^. Even though the binding agent and curing mechanism of the product tested was different of the current study, they had the same bioactive component^75^. The protection in depth is based on the same reasons of ion interaction to tooth structure, as described before^16,65,67,69–71^.

The best approach to completely describe the effects of the treatments on caries lesion prevention would be to associate different methods of analysis. Depth-related characteristics of enamel caries lesions can be described in terms of two main different properties, which are the mechanical aspects and the mineral content^34,55^. Those properties can be measured in vitro by X-ray attenuation and hardness profiles. The transverse microradiography provides a quantitative measure of the mineral content, while cross-sectional microhardness reflects the mechanical resilience of enamel^34,55^. However, the use of a single method, although does not explain completely the phenomenon, also does not invalidate the evaluation performed. The microhardness analysis of the current study provided information about mechanical properties of the lesion, which is clinically relevant, since the collapse of the caries lesion under external forces will lead to the cavitation, and the need of invasive restorative procedures. However, further studies evaluating the mineral profile by transverse microradiography after treatment with the different S-PRG varnish formulations are recommended.

## Conclusion

Under the in vitro conditions, we concluded that all the experimental S-PRG varnishes tested were able to prevent initial enamel demineralization. The higher concentrated products were more effective than 5% sodium fluoride on surface demineralization prevention.

## Data Availability

All data are available from the corresponding author under request.
